# In situ simulation training strengthened bachelor of nursing students’ experienced learning and development process– a qualitative study

**DOI:** 10.1186/s12912-024-01771-w

**Published:** 2024-02-15

**Authors:** Karina Karlsen, Carina Nygård, Lisbeth Gaustad Johansen, Edith Roth Gjevjon

**Affiliations:** 1 UiT The Arctic University of Norway, Harstad, Norway; 2 Lovisenberg University College, Oslo, Norway

**Keywords:** Simulation, Clinical practice, Nursing students, Qualitative design, Content analysis

## Abstract

**Background:**

In advanced clinical learning labs on campus, high-fidelity simulation has become an essential educational approach in the Bachelor of Nursing Education programme. However, simulation while in clinical placement, in situ, is rarely used in Bachelor of Nursing Education. The aim of the present study was to explore how in situ simulation training at a surgical hospital ward, according to Bachelor of Nursing students, influenced their learning and development process.

**Methods:**

A qualitative descriptive study was conducted. Data were collected through individual interviews with a sample of 21 s-year Bachelor of Nursing students who completed 40 in situ simulations during their eight-week clinical placement at a Norwegian University Hospital. Data were analysed using inductive content analysis.

**Results:**

The data analysis generated six subcategories constituting two descriptive categories: building professional confidence and internalising nursing knowledge. Although the students found in situ simulation stressful and uncomfortable for being assessed by student peers, the teacher and preceptor, the process of managing clinical situations in simulation helped build professional confidence. What the students had learned in the simulation was directly transferable to real clinical situations because they were in the hospital setting. The simulation sessions enabled them to connect theoretical knowledge and clinical skills. They could test their skills in a safe environment, performing procedures that made them aware of how their knowledge could be used in real life.

**Conclusion:**

According to the Bachelor of Nursing students’ own experiences, in situ simulation supported the students’ learning process, connected theory and practice and contributed to developing confidence in the performance of clinical skills. Including simulation in clinical practice could prove to be an effective way of teaching and learning clinical skills in nursing regarding resources and learning outcomes.

**Supplementary Information:**

The online version contains supplementary material available at 10.1186/s12912-024-01771-w.

## Introduction and background

Simulation training has become an essential educational approach in Bachelor of Nursing Education. Besides general pedagogical and technological innovation, uncertainties regarding the educational quality of clinical practice call for the further development of simulation-based nursing didactics. In European countries that follow the European Union´s (EU) directive for nursing education, the contexts in which nursing students should be placed and the number of direct care hours are strongly regulated. Students must conduct at least 2,300 h in total, that is, 50% of the curriculum, and be placed in medical and surgical wards, mental health care facilities, children’s and maternity care and home health care [1–3]. However, there are no requirements regarding the quality of the clinical practice, that is, whether the students achieve the required learning outcomes. In addition, nursing students’ exposure to clinical learning situations is often insufficient because of limited available clinical placements and less time spent with patients, especially in surgical contexts, because of reduced hospitalisation time and less invasive inpatient procedures being performed. For educators, it is a common concern that nursing students might be insufficiently prepared for the reality that meets them after graduation [4].

Both clinical practice and simulation training are experiential learning processes, that, together with theoretical learning, are pivotal for achieving the necessary competence, knowledge and skills to provide person-centred and evidence-based safe care for patients [5]. Simulation has been suggested as a means to bridging theory and practice, that is, closing the so-called ‘theory–practice gap’ [6]. The theory–practice gap can be defined as ‘the gap between the theoretical knowledge and the practical application of nursing, most often expressed as a negative entity, with adverse consequences’ [7, p.1]. According to Greenway et al.’s concept analysis [7], the theory–practice gap is mainly caused by practice failing to reflect theory, that theory is perceived as irrelevant to practice or as relational problems between higher education institutions (HEI) and clinical practice.

Simulation training provides a reality-like safe environment for learning, allowing the students to rehearse clinical procedures without risking patient harm while helping integrate theoretical knowledge and clinical skills [8]. Clinical skills are developed and tested ‘in vitro’, preparing nursing students for the nursing profession in a controlled environment [9]. There is evidence to support the idea that simulation training for Bachelor of Nursing students (hereafter also termed nursing students) is beneficial. A recent PhD study explored the combination of clinical practice with on-campus simulation training for first-year nursing students, concluding that simulation training as a supplement to clinical practice increased student satisfaction, self-confidence [5] and learning outcomes [10]. These results are supported by a systematic review of high-fidelity simulations for nursing students [11]. Tamiselvan et al. [12], however, concluded that, although simulation is beneficial, it is essential that nursing students practice clinical skills in an authentic clinical learning environment to integrate theoretical and practical knowledge, skills and competence. Based on the literature and general discussions with peers, one might get the impression that clinical practice and simulation is a question of either-or in Bachelor of Nursing Education.

Typically, in nursing education, high-fidelity simulation takes place in on-campus training facilities, mimicking the context in which the nursing activities take place. Simulation in natural clinical contexts, that is, in situ, is less common. In situ simulation is, according to a recent literature review, mostly used in specialised settings, aiming to educate staff and improve advanced or acute care skills and teamwork to increase patient safety [13]. However, based on their study, Ravik and Bjørk [14] recommend in situ simulation for nursing students, assuming that it would enhance students’ procedural learning and improve skill performance because the simulations take place during clinical placement and not in on-campus simulation facilities. Research on on-campus simulation training in nursing education is substantial, yet less is known about simulation training as an educational approach in a naturalistic clinical context, i.e., in situ. Therefore, the aim of the present study was to explore how in situ simulation training at a surgical hospital ward, here according to Bachelor of Nursing students, influenced their learning and development process.

## Methods

To explore nursing students’ experiences of in situ simulation training during clinical placements in a specialised hospital setting, a qualitative descriptive study was conducted. A qualitative descriptive approach is well suited for studying experiences and perceptions in a naturalistic context [15] as the collected data remain close to the phenomenon, in this regard nursing students’ learning and development process through in situ simulation, during the whole research process [16].

### Setting

In Norway, a Bachelor of Nursing Education is a three-year education programme, with 50% of the time spent in clinical studies under the supervision of onsite registered nurses (RNs). In the current study, it was mandatory for the second-year students to participate in three in situ full-scale simulation sessions during their hospital placement at a surgical unit. Following the International Association for Clinical Simulation and Learning [17], including briefing, training and debriefing, each scenario was carried out as a joint activity with two students and their supervisor, which was facilitated by the first and third authors. To mirror authentic and reliable nursing situations, the simulation session was conducted in an equipped patient room with a living marker, that is, a person rather than a simulation mannequin. The scenarios were relevant to the surgical nursing field and were created in close collaboration with the hospital’s student coordinator. The researchers completed a total of 40 simulation scenarios.

### Sample

A purposive sample of 21 students enrolled in UiT The Arctic University of Norway’s (UiT) second-year nursing programme who completed their mandatory simulation sessions during their hospital clinical placement participated. The students were recruited through the study director with permission from the hospital management. All participants gave informed consent to participate. The sample included both sexes, with a mean age of 26, ranging from 18 to 50. Seventeen participants were women, and four were men.

### Data collection

The data collection took place between May and October 2018. The first, second and third authors each conducted individual interviews with the students after their completion of the eight-week clinical placement. Each interview was held in a meeting room at the hospital or university campus chosen by the participant. The interviews followed a semistructured interview guide developed for this study aiming to capture the students’ learning and development experiences from the in situ simulation sessions (see supplementary file). The students were encouraged to reflect upon their motivations and expectations for their simulation training, their team collaboration and how they coped with challenges and benefitted from a diverse learning environment. The interviews lasted between 30 and 60 min and were digitally recorded and transcribed verbatim. The data material used for analysis consisted of about 168 pages of transcribed text.

### Data analysis

Data were analysed by using inductive content analysis [18]. This method of analysis is widely used in qualitative descriptive research for providing meaningful data-driven descriptions of people’s experiences [19]. The interview transcripts contained manifest and latent descriptions reflecting the participants’ perceptions. Manifest contents are those explicitly stated by the participant, while latent contents are the underlying meanings and assumptions that are expressed indirectly [18]. First, the authors read the transcribed interview text several times to obtain an overall sense of the data. Each author then divided the text into meaning units that were condensed and coded. The codes related to the aim of the study were grouped into tentative subcategories based on their similarities and differences. In the second round of analysis all authors compared and discussed codes and tentative subcategories. Six subcategories were agreed upon after reflection and discussion between the authors and further interpreted and abstracted into two broad descriptive categories: (1) building professional confidence and (2) internalising nursing knowledge. Table 1 illustrates the process of analysis.

**Table 1:** Illustration of the process of analysis, from meaning units to descriptive themes

Meaning units	Condensed meaning units	Code	Subcategories	Descriptive categories
I was a bit stressed, but stress teaches you a lot. As a team leader on a sepsis case, everything halted, and I became irritable and stressed. While I did not feel like I learned much at the time, after I calmed down I realized I learned a lot about myself and the case. (ID 7)	Felt stressed and disliked it. Learned about oneself and the case after calming down.	Learned from stress and learned a lot about oneself.	Managing stress, uncertainty and fears.	Building professional confidence.
I enjoyed being with my fellow students. Throughout this period our relationship has developed, and we have become very close. During the simulations, we discussed the scenarios and pushed each other professionally. After the session, we reflected upon our experiences. (ID 8)	Personal and professional relationships with fellow students were valuable.	Connecting and collaborating with peers contributed to development.	Being part of a team.	
I feel more confident in my line of work after doing these simulations. I have really benefitted from this. Both the knowledge and development of my skills. I have gotten a feeling of empowerment. (ID 7).	After completing simulations, feeling confident and empowered. It was beneficial to increase knowledge and skills.	Confidence and empowerment are built through simulation.	Believing in one’s own capabilities.	
We get to sort out and connect different situations to different things. The theory can be hung on the skills we performed or forgot to perform. That’s how I learned the most. (ID 11)	Experiencing different situations, doing or forgetting things contributed to learning theory and skills.	Learning occurs through experience in different situations	Connecting theoretical knowledge and nursing actions.	Internalising nursing knowledge.
I think that doing simulation in situ is a great thing to do. You get to practice nursing skills without any risk of hurting patients. You can fail and retry without causing any damage. It’s a great opportunity for learning (ID 4).	The ability to attempt and fail while practicing nursing without harming a patient contributes to the learning process	Practicing and learning nursing skills without risking damage.	Risk-free exploration of nursing skills.	
You get to practice different situations that make you safer and make you handle real situations in a better and safer way. And you get to do this in safe environments. (ID1).	Simulating different situations allows the handling of real-life situations to be practiced.	Learning is enhanced by rehearsing different situations.	Learning by doing.	

The subcategories depict the ‘red thread’ across condensed meaning units and codes, while the descriptive categories constitute a comprehensive interpretation of the data [20].

### Ethical considerations

The present study was designed to comply with the Norwegian National Research Ethics Committee’s general guidelines for research ethics, and the Norwegian Agency for Shared Services in Education and Research assessed the research protocol (project number 59,714). Permission to recruit students was obtained from the Head of the Institute of Health and Care Sciences at UiT, as well as the Head Nurse and the department’s Nurse Manager at The University Hospital of Northern Norway (UNN). None of the research team members provided grades to the students attending the study. However, because of the size of the campus and relatively small class of students (*N* = 70), three of the authors knew the students from previous teaching situations. The students in the study were adequately informed about the overall study objectives. Before the interviews, all students provided written and oral consent and were free to withdraw at any time. Anonymity was ensured for all participants. Audio recordings were captured and transcribed verbatim. The data were processed and stored according to UiT’s recommendations.

### Trustworthiness

Trustworthiness in qualitative research is evaluated by considering the following four criteria: credibility, dependability, confirmability, and transferability [21]. Credibility was ensured by a sample including all eligible participants for the study and that all participants, 21 nursing students, were interviewed individually. For dependability, data was collected in proximity to the clinical placement period where the in situ simulation training took place, ensuring that their experiences were recent and consistent when interviewed. To enhance the confirmability of the results, all authors participated in the process of analysis by independently and in collaboration analysing the data and reaching a consensus on the final results. Direct quotes from participants were used to illustrate the findings. Transferability has been facilitated by descriptions of the context, setting, and the selection and characteristics of the participants. Consolidated Criteria for Reporting Qualitative Research (COREQ) [22] was used to ensure quality and transparency during the research process and reporting.

## Results

The overall results show that simulation in a naturalistic setting at a surgical hospital ward was well received by the students, although they described taking steps out of their comfort zones during the clinical placement period. The setting in which they were challenged was, however, considered safe and trustworthy, facilitating their learning and development process. In situ simulation training seemed to help build the students’ professional confidence and internalise their nursing knowledge. Below, quotations illustrating the descriptive categories are identified with participant numbers (ID 1–21).

### Building professional confidence

Most students expressed stress, uncertainty and fear in their initial simulations because they were unfamiliar with the learning method. They sensed a lack of knowledge and feared failing to master the simulation because of being judged by student peers, teachers and preceptors, illustrated by the following excerpt:

I was so stressed. I started to think; do I have enough knowledge? Am I good enough for the nurses? I knew we were not tested, but I was so stressed that it felt as we were. (ID 10).

Although experiencing stress, uncertainty and fear, students reported that frequent simulation training during their placement helped them feel more confident and competent. One student stated the following:

It is understandable that this can be stressful, so simulation must be rendered harmless. I noticed that no one seemed nervous during the final simulations. It wouldn’t be so scary if we did this more often. (ID 9).

Repeated simulations contributed to mitigate stress and uncertainty and gave them a sense of mastery as well as controlling the fear of failing.

### Managing stress, uncertainty, and fear

Although a minority of the students remained stressed throughout the simulation sessions, they described this as a ‘positive stress’, explaining that, even though they felt uncomfortable in the moment, this improved learning as they became better acquainted with their weaknesses and reflected critically upon it afterwards:

I was a bit stressed, but stress teaches you a lot. As a team leader on a sepsis case, everything halted, and I became irritable and stressed. While I did not feel like I learned much at the time, after I calmed down I realized I learned a lot about myself and the case. (ID 7).

These experiences enabled them to become more comfortable with the possibility of facing risks and making mistakes, hence ultimately gaining more confidence in their ability to succeed in actual clinical practice. Thus, this enhanced their problem-solving skills and their ability to make rapid decisions under pressure in a controlled environment. One student said the following:

I have applied these experiences to real clinical situations, and I manage to maintain calm in stressful situations. (ID 11)

Additionally, the students expressed that constructive counselling from their preceptors and teachers was essential for managing stress and uncertainty. The teachers’ presence and feedback comforted, reassured and confirmed their knowledge. This motivated and prompted them to initiate and engage in professional discussions with other team members (student peers, preceptors) and teachers. The presence of preceptors encouraged most students to keep going by giving them hints and helped them through if they stopped. Some felt intimidated by the RNs because they were too critical and judgemental in their comments. This put the students off from asking questions in fear of revealing their insufficient knowledge. One student claimed the following:

I felt along the way that we were assessed by the nurses who were there. Didn’t like it that much. For my part, we could have been without them (ID 17).

### Being part of a team

All the students agreed that simulation training with fellow students they knew well increased their comfort levels. This allowed a more relaxed learning environment, which enabled students to form closer bonds with their peers. Mutual help and encouragement alleviated the induced stress and uncertainty, as this student memorised:

I enjoyed being with my fellow students. Throughout this period our relationship has developed, and we have become very close. During the simulations, we discussed the scenarios and pushed each other professionally. After the session, we reflected upon our experiences. (ID 8)

The following student’s quote sums up a benefit of the in situ simulation training:

I feel more confident in my line of work after doing these simulations. I have really benefitted from this. Both the knowledge and development of my skills. I have gotten a feeling of empowerment. (ID 7)

As illustrated by the student in the above quote, the in situ simulation training seemed to not only affect how the students developed their confidence, but it also strengthened their nursing knowledge.

### Internalising nursing knowledge

The students were able to practice in a safe environment, trying out different treatments and procedures that made them aware of how their knowledge could be used in a real-life setting:

I had a patient who was ready to be discharged from the hospital. She seemed fine at the time. Suddenly, she felt ill and was in a lot of pain. I managed to take her vitals and get an overview of the situation myself before reaching out to my preceptor. It felt like I had developed a kind of spinal reflex. (ID 9)

The quote above illustrates that the students experienced situations where they were being able to use their knowledge without having to validate their actions with their preceptors or by reading textbooks. They demonstrated that theoretical knowledge and nursing actions were connected.

### Connecting theoretical knowledge and nursing actions

All the students expressed that they had learned to connect theoretical knowledge and nursing actions from simulation in practice, as illustrated in the following statement:

We get to sort out and connect different situations to different things. The theory can be hung on the skills we performed or forgot to perform. That’s how I learned the most. (ID 11)

The students acknowledged that, by simulating different acute cases, they managed to increase their knowledge and competency. They learned that, by doing procedures repeatedly, they were able to transfer their theoretical knowledge to real patients in the same context. One of the students said the following:

By simulating a case where the standardised patient had sepsis, I was able to identify that a real patient was septic a few days later. I knew what to do! (ID 19)

Simulating together with student peers and RNs working in the department was seen as a way to connect theoretical knowledge with nursing actions through repeatedly rehearsing real-life situations as a team:

When you simulate together with your classmates and the nurses, you get to know them and get to do nursing skills with them that you haven’t done before. In that way, we learn to trust each other better. (ID 9)

The students expressed that in situ simulation allowed them to apply theoretical knowledge to practical situations through exploring their skills in collaboration with the members of the team in a safe environment.

### Risk free exploration of nursing skills

The students felt that being able to practice their knowledge and skills in a safe environment helped the learning process:

I think that doing simulation in situ is a great thing to do. You get to practice nursing skills without any risk of hurting patients. You can fail and retry without causing any damage. It’s a great opportunity for learning. (ID 4)

The students expressed that, if they felt drilled enough, this would tend to create a sense of security for them, so they then feel more prepared:

You get to practice different situations that make you safer and make you handle real situations in a better and safer way. And you get to do this in safe environments. (ID1)

The students expressed that the hands-on learning activities strengthened their learning outcomes.

### Learning by doing

The students were explicit in their experience of being able to connect theory and practice through practical rehearsal of nursing skills.

Connecting theoretical knowledge to practice. I think that this should be mandatory for everyone who studies nursing. I feel a calmness that I’ve gained from the simulations. More security in myself. I can’t get this with just reading the theory. (ID 19)

The following quote illustrates the students’ satisfaction with in situ simulation training and expresses considerable faith in the method:

I see the value of the simulation and feel the effect of it. I think we’d have a different health care system out there if everyone had been practicing simulation. (ID 5)

According to the students, in situ simulation training supported their learning process regarding clinical nursing skills and knowledge, as well as contributing to positive development in becoming confident in the performance of their skills.

## Discussion

The aim of the present study was to explore how in situ simulation training of Bachelor of Nursing students at a hospital surgical ward influenced the students’ learning and development process.

The overall findings indicate that in situ simulation is an effective and feasible teaching and learning strategy. On-campus high-fidelity simulation is resource-demanding and costly, and often requires modern, high-tech and spacious facilities. Therefore, in situ simulation may be considered an alternative teaching method—not a replacement for on-campus simulation but a method that can expand the repertoire of nursing didactics. High-fidelity simulation mimics realistic clinical situations and, therefore, has an indisputable positive effect on learning outcomes [23]. In situ simulation takes place in a *real* clinical context, giving students the opportunity to use the knowledge and skills they gained during the simulation directly in the ward, in real-life patient situations. A recent review [13] demonstrated that in situ simulation among health professionals contributes to increased confidence levels, clinical orientation and preparedness, as well as improved understanding of roles and enhanced patient outcomes. Our findings suggest that these results are also true for students.

Our participants stated that performing simulation in situ at the hospital department where they were undergoing their clinical practicum together with their peers, preceptors and teachers increased their feeling of stress. Their described experiences of stress were, to a certain extent, similar to Simpson and Sawatzky’s [24] description of *clinical placement anxiety* as ‘a vague perceived threat to a student’s goals or expectations’ (p. 5) that might negatively affect the student’s learning outcomes. According to our participants’ responses, the opposite may be assumed with in situ simulation, in that rather than anxiety with negative consequences, they described a feeling of ‘positive stress’ benefitting their motivation and coping ability. Selye [25] defined stress not only as something negative or damaging (i.e., distress) but also, at times, as something positive (i.e., eustress) [25], such as adapting to a situation and context, as well as anticipating and handling future challenges [26]. Hence, the students said that although being watched and assessed by their preceptor was stressful for them, it also boosted their confidence and stimulated their learning process [9, 27].

Based on our study and previous knowledge, it is safe to assume that students’ understanding of and confidence in clinical situations and contexts can increase when they share learning experiences with their peers and with experienced RNs. A recent review [28] indicated that participatory teaching methods have a positive effect on nursing students’ motivation to learn. It may also be presumed that such methods create conditions for developing the professional competence of both students and RNs, which, in turn, will attract new RNs and mitigate experienced RNs’ intentions to leave. The learning process in in situ simulation is similar to that in the research-based pedagogical peer-learning model [29, 30], which has been found to build up experience, understanding and knowledge through systematic interactions between peers when collaborating in teams, supporting and learning from and with each other [31]. The outcomes and benefits of peer learning are increased confidence, self-efficacy, competence and clinical skills, as well as reduced stress and anxiety [32, 33]. Our findings on the effects of in situ simulation training on nursing students’ learning are in line with the aforementioned outcomes of peer learning. However, our study was descriptive; therefore, we can at this point only presume that the outcomes of in situ simulation training are similar to those of peer learning. Nevertheless, such outcomes would be relevant variables in future studies on the effects of in situ simulation training.

Nursing is a complex activity that combines knowledge, evidence and technical skills with compassion, person-centredness and relational skills. This complexity complicates the teaching and learning process, hence, requiring structured and comprehensive teaching and learning methods [34]. Yet, challenging conditions in clinical contexts, such as time pressure, rapid change of plans, varying workloads and patients’ needs during a workday, might hinder satisfactory supervision of nurses, which, in turn, might negatively affect students’ learning and development process. Based on our findings, we suggest that pre-planned, systematic and structured clinical training, developed collaboratively between preceptors, teachers and students, can address these challenges.

Greenway et al. [7] have identified relational problems between universities and clinical practice as an attribute of the *theory–practice gap*, suggesting that ‘applying theory in context-specific and workable ways’ should be prioritised [7, p. 4]. The theory–practice gap can be understood as the mismatch between what students learn during their education (theory) and in the real world (practice), as well as between students’ expectations and experiences. In reference to this gap, we believe that in situ simulation is indeed a workable way of applying theory to students’ practical skills training in context. Moreover, hands-on collaboration between students and teachers from the HEI and preceptors from clinical practice during a simulation might mitigate the aforementioned relational problems, creating joint teaching and learning opportunities for future RN colleagues.

A deliberate combination of on- and off-campus theoretical teaching activities, clinical training and simulation is necessary to achieve the required level of nursing competence [34]. Our participants stated that they were able to connect theoretical knowledge and clinical skills in consultation and dialogue with their teachers and preceptors. Internalised nursing knowledge and skills are pivotal for the early detection of patients’ health deterioration, for rapid response and for safeguarding of patient safety. Some of our study participants gave examples of their success in detecting symptoms and signs of patient health deterioration, such as in recognising sepsis, which demonstrates that well-planned and executed simulation supports students in learning how to manage deteriorating patients [35]. A desired outcome of success in applying theory to practice is internalised nursing knowledge—that is, nursing knowledge that becomes part of the nursing student’s nature.

### Strengths and limitations

The present study had a qualitative descriptive design, and the findings may not represent the views and experiences of all nursing students undergoing in situ simulation. Moreover, the participants were recruited from the same university, which might limit the transferability of the findings. However, the sample consisted of all 21 students undergoing an eight-week clinical practice, including three full-scale simulations at a surgical ward during. The relatively large sample of students interviewed individually enabled data spanning different perspectives and experiences. Even though the study took place in 2018, the data are still considered relevant. In situ simulation in nursing education is, as far as we have identified in the literature, seldom used compared with on-campus high-fidelity simulation. In fact, in situ simulation has been called for in a recent publication [14].

Three of the authors (KK, CN and LGJ) are nurses with extensive clinical backgrounds in caring for hospitalised patients as well as experienced nurse educators. Close familiarity with the field of study might implicitly lead to biased interpretations, yet on the other hand, the unique insight makes it possible to identify patterns less visible to an outsider [36]. The fourth author (ERG) has limited experience within the clinical context of the study as well as simulation as a teaching method. ERG is a professor of nursing with extensive experience in giving theoretical lectures and conducting research within nursing practice. She participated in and supervised the analytical process and had a leading role in the final analysis of the data, as well as interpretations of the findings. The whole team met regularly to reflect on the findings until a consensus was reached.

## Conclusion

Doing simulation in a real clinical context and being assessed by student peers, the teacher and preceptor could lead to a feeling of stress and uncertainty among students. At the same time, the in situ simulations in a safe and authentic setting helped build professional confidence. Through in situ simulation, the students experienced being able to connect theory and practice, which can be interpreted as internalising nursing knowledge. In situ simulation supported the students’ learning process and contributed to developing confidence in performing clinical skills. Including simulation in clinical practice could prove to be an effective way of teaching and learning clinical skills in nursing. Our findings indicate that the learning outcomes are considerable, and few extra resources are required regarding in situ simulation. No specialised facilities or equipment is needed; the setting is real-life, and the clinical situations are authentic. Hence, in situ simulation can easily be implemented in nursing education. Finally, our findings are suited for informing interview guides or observation protocols or yielding hypotheses for future research.

### Electronic supplementary material

Below is the link to the electronic supplementary material.

Supplementary Material 1

## Data Availability

The interview transcripts (in Norwegian) are available upon request.

## Abbreviations

RN: Registered nurse
UNN: The University Hospital of Northern Norway
UiT: The Arctic University of Norway
HEI: Higher Education Institution
