# Supplementary material for: In situ simulation training strengthened bachelor of nursing students’ experienced learning and development process– a qualitative study
Source: BMC Nurs. 2024 Feb 15;23:121. doi: 10.1186/s12912-024-01771-w (PMC10870516; doi:10.1186/s12912-024-01771-w)
Supplement: Supplementary file 1 — Supplementary Material 1 [file 12912_2024_1771_MOESM1_ESM.docx]

**Interview guide – In situ simulation**

**Topic:** Nursing students’ experiences of simulation in surgical practice (in situ).

**Framing**

- Informal conversation (2-5 min)
- Repeat information about the project and research questions (bring written information and consent form)
- Repeat the purpose of the interview
- Repeat information about what will the interview be used for
- Repeat information about confidentiality and confidentiality
- Obtain consent for the use of audio recordings
- Ask if the participant has any questions before commencing the interview
- Register background data: sex, age
- Start audio recording

**Core questions with probes**

- What are your thoughts about simulation as a teaching/learning method?
- Tell me about how you experienced doing simulation while in clinical practice
  - Did you learn anything? Elaborate
- Was this clinical practice, that included simulation, any different from a normal clinical practice without simulation? How or why?
- What did you experience as positive about using simulation in practice?
- What did you experience as negative about using simulation in practice?
- How did you experience simulating together with fellow students and your preceptor?
  - Positive experiences, negative experiences, or both?
- Has your own perception of simulation changed during this clinical practice?
- Would you recommend more simulation during clinical practice, as it is, or less? Why?
- Is there anything I should have asked you that we didn’t ask, or is there anything you would like to add?

**Closing the interview**

- Summary of the interview
- Clarifications if misunderstandings have occurred
- Remember to thank the participant for attending the interview

**Stop audio recording**
